# Predictors of real‐world adherence to prescribed home exercise in older patients with a risk of falling: A prospective observational study

**DOI:** 10.1002/agm2.12270

**Published:** 2023-09-27

**Authors:** Bernadine Teng, Sjaan R. Gomersall, Anna L. Hatton, Asaduzzaman Khan, Sandra G. Brauer

**Affiliations:** ^1^ School of Health and Rehabilitation Sciences The University of Queensland Brisbane Queensland Australia; ^2^ Health and Social Sciences cluster Singapore Institute of Technology Singapore Singapore; ^3^ School of Human Movement and Nutrition Sciences The University of Queensland Brisbane Queensland Australia

**Keywords:** accidental falls, aged, exercise, patient compliance, rehabilitation

## Abstract

**Objectives:**

Using a multi‐ethnic Asian population, this study assessed adherence to prescribed home exercise programs, explored factors predicting adherence, and evaluated whether home exercise adherence was associated with physical activity.

**Methods:**

A prospective cohort study was conducted in 68 older adults (aged ≥65 years) from two geriatric outpatient clinics in Singapore, who were receiving tailored home exercises while undergoing 6 weeks of outpatient physical therapy for falls prevention. Adherence was measured as the percentage of prescribed sessions completed. Predictor variables included sociodemographic factors, clinical characteristics, intervention‐specific factors, and physical and psychosocial measures. Multivariable linear regressions were performed to develop a model that best predicted adherence to prescribed exercise. Physical activity levels, measured by accelerometry, were analyzed by cross‐sectional univariate analysis at 6 weeks.

**Results:**

The mean adherence rate was 65% (SD 34.3%). In the regression model, the number of medications [*B* = 0.360, 95% CI (0.098–0.630)], social support for exercising [*B* = 0.080, 95% CI (0.015–0.145)], and self‐efficacy for exercising [*B* = −0.034, 95% CI (−0.068–0.000)] significantly explained 31% (*R* ^2^ = 0.312) of the variance in exercise adherence. Older adults with better adherence took more steps/day at 6 weeks [*B* = 0.001, 95% CI (0.000–0.001)].

**Conclusions:**

Low adherence to home exercise programs among older adults in Singapore, emphasizing the need for improvement. Counterintuitively, older adults with more medications, lower exercise self‐efficacy, but with greater social support demonstrated higher adherence. Addressing unmet social support needs is crucial for enhancing adherence rates and reducing fall risks.

## Graphical abstract

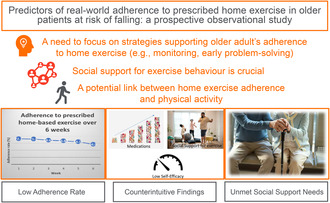

## 1 INTRODUCTION

It is well established that home exercise programs can reduce falls rate and falls risk.^1^ Studies have shown that higher doses of exercise, specifically 3+ h per week of balance‐specific training, can have greater effect on reducing the rate of falls in older adults at risk of falling.^2^ One common approach to ensure sufficient dose is to prescribe unsupervised home exercise; however, achieving adherence to home exercise can be challenging. Adherence refers to the extent to which an individual's behavior corresponds with agreed recommendations from a health care provider.^3^ Non‐adherence to treatment leads to more frequent falls, greater use of health care resources from falls‐related injuries,^4^ and less success in decreasing falls.^5^

The efficacy of individualized home‐based exercise has been clearly demonstrated in randomized controlled trials.^1^, ^6^ However, adherence to prescribed exercise in real‐world settings has not been well characterized. Reported adherence to prescribed home‐based exercise in the literature has varied, with one review reporting a rate of 21%,^7^ and is known to decline over time.^8^ While there is no consensus on the definition of adequate adherence at present,^9^ overall adherence is typically inadequate.

Limited and inconsistent findings exist regarding predictors of adherence to falls‐prevention home exercises in older adults. One study found that taking fewer medications was associated with better adherence.^10^ Another study showed that better cognitive function and functional mobility predicted greater adherence to the Otago Exercise Program.^11^ Additionally, higher levels of physical activity predicted increased exercise adherence.^12^ Understanding the association between adherence to home exercise programs and subsequent physical activity levels is important.^6^

Given the limited information on adherence to prescribed exercise, the present study aimed to: (1) evaluate real‐world adherence to prescribed home exercise; (2) explore factors predicting adherence to prescribed home exercise programs designed to reduce falls risk/falls rate in older adults during the initial 6 weeks of rehabilitation; and (3) determine any association between adherence to home exercise and physical activity levels at 6 weeks.

## 2 METHODS

### 2.1 Design

An observational cohort study was conducted between May 2019 and March 2020 with assessment time points of T0 (baseline) and T6 (6 week follow up). Participants were recruited through convenience sampling from two major hospital‐based geriatric outpatient clinics in Singapore. Participants were involved in a 6‐week, individually tailored home exercise intervention while undergoing outpatient physical therapy for falls prevention delivered in public hospitals. Ethical approval was obtained from The Singapore National Healthcare Group – Domain Specific Review Board (2018/01372) and The University of Queensland Human Research Ethics Committee (HREC/2019001991). All participants provided written informed consent prior to enrolment in the study.

### 2.2 Participants and setting

Participants aged 65 years or above, deemed at increased risk of falls, were included in the study. Increased risk was defined by attendance at an outpatient falls and balance clinic (with/without history of falls) and assessment by a geriatrician and/or physiotherapist in hospital‐based geriatric outpatient clinics. Exclusion criteria comprised nursing home residency and cognitive impairment hindering the ability to provide informed consent or understand study procedures. Cognitive status was evaluated during initial screening using the Short Portable Mental Status Questionnaire, considering cognitive impairment if there were ≥3 errors.^13^

### 2.3 Adherence measure

Participants were asked to complete a paper‐based exercise diary recording each time they completed a session of their individually prescribed home exercise program over the 6‐week treatment period. Adherence was defined as percent completion of prescribed home exercises over 6 weeks, following the commonly reported definition in the literature.^14^ Adherence rate was calculated by way of the total number of home exercise sessions completed as a proportion of that prescribed each week from T0 to T6, to determine each participant's overall completion rate (%), and completion rate per week (%).

### 2.4 Potential predictors

Selection of potential predictors was informed by previous literature and constructs of the COM‐B behavior change model.^15^ Thirty‐one variables were selected (Appendix S1). Sociodemographic and clinical characteristics were collected through a self‐report questionnaire administered. Intervention‐specific variables were obtained from physical therapy records, including home exercise prescription details and follow‐up information. Physical and psychological abilities were measured using validated measurements such as the 30‐s chair stand test,^16^ the Timed Up and Go test,^17^ the Falls Efficacy Scale‐International,^18^, ^19^ and the Self‐Efficacy for Exercise Scale.^20^ Social opportunity influences were measured using the validated 13‐item Social Support for Exercise Behaviors survey that assesses perceived social support for exercise during the past 3 months from family and friends,^21^ and a 3‐item short form of the Revised University of California Los Angeles Loneliness Scale was used.^22^ Physical activity was measured using the Actigraph GT3X accelerometer (ActiGraph) that was worn at T6 (6 weeks from baseline) and the Phone‐FITT survey.^23^

### 2.5 Data analysis

Baseline characteristics were analyzed using descriptive statistics. Categorical variables were described as percentages and continuous variables as mean (SD). It has been proposed that selecting variables begins with univariate analyses^24^; we assessed all 31 potential predictors (Appendix S1) using univariate linear regression. Potential predictor variables with a univariate association with the primary outcome at a significance level of *p* < 0.15 were retained as candidate predictor variables for the multivariate linear regression model. A higher significance level (*p* < 0.15 compared to *p* < 0.05) was used to select variables so that important variables relevant to the outcome were not missed, and to avoid deleting less significant variables that had practical or clinical significance.^25^ To obtain the final multivariable linear regression model for adherence, the alpha level was set at 0.05 and backward elimination was used. Percent exercise adherence data were not normally distributed and were therefore transformed using a square root transformation. Preliminary analyses were performed to ensure there were no violations of the assumptions of normality, linearity, homoscedasticity, and multicollinearity. Cases were excluded if standardized residuals exceeded ±2. SPSS (v26; IBM Corp) was used for all statistical analyses.

We conducted a cross‐sectional analysis for physical activity measured by accelerometer at T6 using univariate linear regression. Actigraphs were initialized, data downloaded, and variables (i.e., steps count, sedentary, light/moderate/vigorous activities) were derived using the Actigraph proprietary software ActiLife (v6.13.4, ActiGraph). Raw data were integrated into 60‐s epochs for analysis. Minimum wear time was defined as 10 h per day, and a minimum number of 4 valid days (with at least 1 weekend day) was required for inclusion in data analysis.^26^ Non‐wear time was considered as 60 min of consecutive zeros. Total step counts (average steps/day) and minutes (min/day) spent in sedentary, light, moderate, and vigorous physical activity were derived using cut points (counts/minute) for: sedentary 0–99; light ≥100–2019; moderate 2020–5998; vigorous ≥5998; moderate‐to‐vigorous intensity ≥2020 activity.^27^, ^28^

## 3 RESULTS

### 3.1 Participants

Seventy‐two patients were initially recruited, but four participants withdrew prior to T0. However, data collection was ceased in March 2020 due to COVID‐19 restrictions. As a result, data analysis was conducted for 68 participants (*n* = 68; 78 ± 6.7 years) (Table 1).

**TABLE 1:** Characteristics of the study sample.

Sociodemographic characteristics	*n*	Value
Age (years)	68	78.9 ± 6.8
Body mass index (kg/m^2^)	65	25 ± 6.1
Gender (female), *n* (%)	68	37 (54.4)
Marital status (married), *n* (%)	68	64 (94.1)
Ethnicity		
Chinese, *n* (%)		54 (79.4)
Indian, *n* (%)		4 (5.9)
Malay, *n* (%)		9 (13.2)
Other, *n* (%)		1 (1.5)
Education level	66	
No formal education, *n* (%)		19 (27.9)
Primary, *n* (%)		14 (20.6)
Secondary, *n* (%)		27 (25)
Tertiary, *n* (%)		16 (23.5)
Living situation	68	
Alone, *n* (%)		5 (7.4)
With spouse/children, *n* (%)		54 (79.4)
With other family members/friends, *n* (%)		9 (13.2)
Multigeneration household (≥3), yes, *n* (%)	67	23 (34.3)
*Clinical characteristics*		
Visual impaired (yes), *n* (%)	68	18 (26.5)
Hearing impaired (yes), *n* (%)	68	17 (25)
Functional mobility	68	
Independent, *n* (%)		52 (76.5)
Non‐independent, *n* (%)		16 (23.5)
Falls history in the last 12 months (yes), *n* (%)	67	52 (76.5)
Number of falls	66	
None, *n* (%)		14 (20.6)
Once, *n* (%)		27 (39.7)
Twice, *n* (%)		7 (10.3)
Three or more, *n* (%)		18 (26.5)
Pain (yes), *n* (%)	61	24 (35.3)
Use of walking aid (yes), *n* (%)	68	36 (52.9)
Number of medications	65	5.0 ± 3.1
Number of chronic conditions	68	2.4 ± 1.1
*Psychosocial variables*		
Short Falls Efficacy Scale‐International (score/28)	68	17.4 ± 6.4
Self‐Efficacy for Exercise Scale (score/90)	67	35.8 ± 22.3
Social support for Exercise Behaviors (score/100)	66	29.2 ± 11.0
Short R‐UCLA Loneliness Scale (score/9)	67	4.2 ± 1.4
*Physical performance*		
30‐s chair stand (repetitions)	53	6.6 ± 5.0
Timed up and go test (seconds)	54	23.2 ± 19.3
Phone‐FITT (sum score)	66	24.2 ± 15.4
*Intervention‐specific variables*		
Number of different home exercises prescribed	60	3.4 ± 1.2
Frequency of onsite follow‐up (total visit over 6 weeks)	64	2.2 ± 1.6
Time between T0 and the first onsite follow‐up (weeks)	66	3.1 ± 2.5
Balance component in prescribed exercise (yes), *n* (%)	61	49 (72.1)
Strength component in prescribed exercise (yes), *n* (%)	61	42 (61.8)
Functional component in prescribed exercise (yes), *n* (%)	61	31 (45.6)
Combined exercises (yes), *n* (%)	61	32 (47.1)

*Note*: Data are presented as mean ± SD unless indicated otherwise, e.g., *n* (%). Max *n* = 68. All variables were collected at baseline except intervention‐specific variables were collected during the intervention.

### 3.2 Adherence rate

**FIGURE 1:**
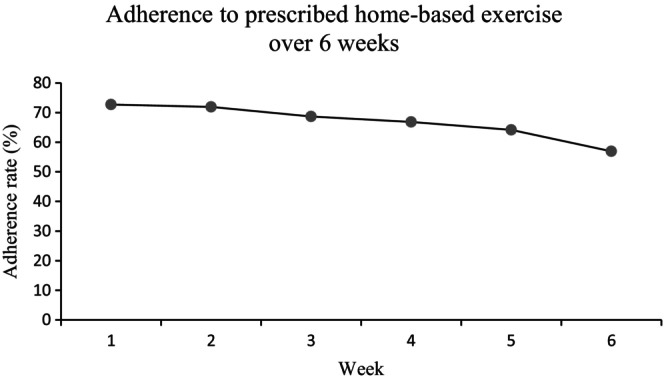
Mean adherence rate over 6 weeks.

**FIGURE 2:**
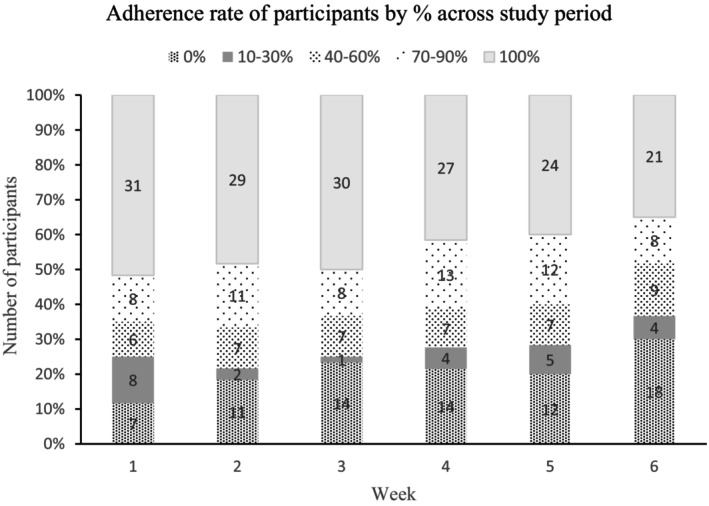
Adherence rate of participants (*n* = 60) across study period.

Out of the initial 68 participants, 60 were included in the analysis, as 8 participants did not return their exercise diary. The mean adherence to the 6‐week home exercise program was 65% (SD: 34.3%). Among the participants, 12 (20%) were fully adherent, completing all prescribed exercise sessions while 5 (8%) did not initiate or perform the prescribed exercises. Notably, the mean adherence rate exhibited a downward trend over the 6‐week period (Figure 1), and Figure 2 illustrates the increasing number of participants with non‐adherence as the weeks progressed and vice versa.

### 3.3 Predictive factors

Univariate linear regression analysis identified two variables to be significantly (*p* < 0.05) associated with exercise adherence; number of medications [coefficient 0.324, 95% CI (0.064, 0.583)] and no use of walking aid [coefficient 1.817, 95% CI (0.272, 3.362)]. Participants with no formal primary education (*p* = 0.053), performed well in the 30 s chair stand test (*p* = 0.076), or had good social support for exercise behavior (*p* = 0.071) were also likely to adhere to exercise prescribed, but these relationships did not reach statistical significance. The results of the univariate analyses are provided in Table 2. Eight out of 31 variables (gender, education level, number of medications, use of walking aid, functional component in prescribed exercise, 30 s chair stand test, social support for exercise, self‐efficacy for exercise) were included in the multivariable linear regression because their *p* values satisfied the a priori threshold of <0.15. However, two variables (use of walking aid, functional component of exercise) were thereafter removed due to collinearity with performance of the 30 s chair stand test (indicated by the variance inflation factor >1.35). In total, six variables were entered into the multiple regression model analysis; through the backward elimination process, three variables were removed.

**TABLE 2:** Univariate linear regression between exercise adherence and sociodemographic, clinical, intervention‐specific, physical, psychosocial variables (*n* = 60 for all variables except 30‐s chair stand, *n* = 45, and timed up and go, *n* = 47).

Potential predictors	*B*	95% CI	*p* Value
*Sociodemographic*			
Gender (female)	1.186	−0.408–2.780	0.142
Age (years)	−0.002	−0.122–0.118	0.975
Body mass index (kg/m^2^)	−0.015	−0.150–0.120	0.823
Ethnicity – reference is other			
Chinese	−4.588	−10.906–1.731	0.151
Malay	−4.649	−11.336–2.038	0.169
Indian	−4.854	−12.077–2.369	0.184
Education level (no formal to primary)	−1.542	−3.108–0.023	0.053
Marital status (never married)	1.645	−2.037–5.328	0.375
Living situation – reference is with other family members/friends			
Alone	1.419	−2.078–4.917	0.420
With spouse/children	0.118	−2.167–2.404	0.918
Multigeneration (no)	0.588	−1.086–2.244	0.480
*Clinical variables*			
Number of medications	0.324	0.064–0.583	0.015*
Falls history (no)	−0.267	−2.228–1.693	0.786
Number of falls – reference is none			
Once	−0.299	−2.458–1.860	0.782
Twice	0.407	−2.532–3.346	0.783
Three or more	1.057	−1.284–3.398	0.370
Use of walking aid (no)	1.817	0.272–3.362	0.022*
Hearing impairment (no)	−0.206	−2.032–1.621	0.822
Visual impairment (no)	0.760	−1.022–2.542	0.397
Functional mobility (non‐independent)	−1.274	−3.207–0.659	0.192
Pain (no)	0.197	−1.465–1.858	0.814
Number of chronic conditions	−0.057	−0.790–0.676	0.876
*Intervention‐specific variables*			
Frequency of onsite follow‐up (total number visit over 6 weeks)	−0.284	−0.781–0.212	0.257
Time between T0 and first onsite follow‐up (weeks)	−0.043	−0.362–0.276	0.790
Number of different home exercises	−0.440	−1.087–0.206	0.178
Home exercise prescription components			
Strength component (no)	0.422	−1.337–2.182	0.633
Balance component (no)	−1.089	−3.157–0.980	0.296
Functional component (no)	1.233	−0.352–2.817	0.125
Combination of exercises (no)	−0.144	−1.764–1.475	0.859
*Physical variables*			
30‐s chair stand test	0.174	−0.019–0.367	0.076
Timed up and go test	−0.012	−0.058–0.034	0.599
Phone‐FITT	0.012	−0.040–0.065	0.640
*Psychosocial variables*			
Social support for exercise behaviors	0.066	−0.006–0.138	0.071
Self‐Efficacy for Exercise Scale	−0.027	−0.062–0.009	0.136
Short Falls‐Efficacy Scale‐International	−0.044	−0.175–0.086	0.499
Short R‐UCLA Loneliness Scale	0.056	−0.534–0.646	0.850

*Note*: *B* = unstandardized coefficient.

Abbreviation: CI, confidence interval.

* *p* < 0.05.

The final multivariable regression model (Table 3) included three significant predictors (number of medications, social support for exercise, and self‐efficacy for exercise) that explained 31% (*R* ^2^ = 0.312) of the variance in exercise adherence *F* (3, 40) = 6.045, *p* = 0.002 (Table 3). A comparison was made between baseline data for those participants included (*n* = 44) and not included (*n* = 16) in the final model. No statistically significant differences were found between groups with respect to characteristics in Table 1 (*p* ≤ 0.05) (Appendix S2).

**TABLE 3:** Results of the multivariate linear regression analysis for predicting adherence to prescribed home exercise (*n* = 44).

Independent variables	*B*	*β*	*p* Value	95% CI for *B*
(Constant)	1.696		0.221	−1.062 to 4.455
Number of medications	0.423	0.416	0.003*	0.153 to 0.693
Social support for exercise	0.081	0.327	0.017*	0.015 to 0.148
Self‐efficacy for exercise	−0.034	−0.265	0.051	−0.068 to 0.000

*Note*: Adjusted *R* ^2^ = 0.260; *B*, regression coefficient; *β*, standardized coefficient.

* Statistical significance = 0.05.

### 3.4 Physical activity

Out of 68 participants, valid accelerometry data were available for 48 participants. Fourteen participants had missing data for various reasons: eight did not complete the study, three misplaced their accelerometer, three refused to wear the accelerometer, and six did not meet minimum wear time criteria. The physical activity and sedentary behavior of the 48 participants at T6 are presented in Table 4. In the cross‐sectional univariate analysis at T6, average steps per day within the physical activity factors was found to be significantly associated with exercise adherence. This association explained a significant proportion of variance in adherence scores, with an *R* ^2^ value of 0.132, *F* (1, 45) = 6.848, *p* = 0.012.

**TABLE 4:** Average time spent per day in sedentary behavior and participating in physical activity (*n* = 48).

Outcome	Mean	SD	Range
Steps	2022	1764	141–8080
MVPA (min)	4.7	5.74	0.37–36.68
Light (min)	100.9	58.11	9.70–221.15
Moderate (min)	4.6	5.71	0.37–36.52
Vigorous (min)	0.1	0.13	0.00–0.54
Sedentary (hour)	11.49	1.65	8.64–17.64
Wear time (hour)	13.25	1.74	10.80–21.53

Abbreviations: MVPA, moderate and vigorous physical activity; SD, standard deviations.

## 4 DISCUSSION

This study provides valuable insights into real‐world adherence patterns and factors contributing to home exercise adherence of older adults attending geriatric outpatient clinics in two of the largest public hospitals in Singapore. Counterintuitively, older adults who take more medications, have greater social support, but lower self‐efficacy for exercise, demonstrate greater adherence to home exercise programs. Further, we provide evidence that adherence to home exercise programs is positively correlated with physical activity (step count/day) at 6 weeks.

Our findings highlight suboptimal adherence to home exercise programs in older adults at risk of falls, with only 20% fully adherent, similar to previous studies in Western countries.^7^ Furthermore, we observed a decline in adherence over time, consistent with trends seen in older adults with initially high adherence rates.^8^ This suggests the need for effective strategies to monitor and support exercise adherence in older adults, coupled with early intervention and collaborative efforts to overcome barriers and promote successful outcomes.

At baseline, we explored several possible predictive measures, but these variables were not able to explain most of the variability (69%) in exercise adherence. It is likely that unknown factors play a greater role in the prediction of exercise adherence in older adults at risk of falling and were not measured in this study. Factors such as depression,^29^ cognitive function (e.g., executive function),^11^ and health status^30^ were not measured in the current study but have previously been related to exercise adherence. The use of self‐report for measuring exercise adherence introduces potential variability in the accuracy of the dependent variable data, highlighting the complex and multifactorial nature of exercise adherence.

Contrary to previous studies,^10^, ^31^, ^32^ baseline medication use predicted positive home exercise adherence, possibly due to differences in study design, sample characteristics, and attitude towards health instructions.^10^ Tailoring exercise programs to specific populations is crucial due to adherence complexity. Further investigation is needed to explore the influence of medication count on exercise adherence and its impact on physical activity levels, considering medications such as benzodiazepines and anticholinergics that hinder functional status in older adults.^33^ Yet, enhancing exercise capability may not translate to increased physical activity levels.^34^ Motivation for health improvement may vary based on medication usage, with individuals taking fewer medications showing less concern, while those with multiple medications making greater efforts, aligning with the Health Belief Model.^35^

Adherence to prescribed exercise was positively associated with social support, aligning with the concept that social support can enhance exercise behavior by strengthening self‐efficacy and expectations regarding exercise benefits.^36^ This finding is consistent with previous review on adherence to home‐based exercise and social support.^37^ However, it is concerning that the average scores for social support in our study (family: 32%, friends: 26%) were relatively low. Despite a high proportion of older adults living with their families in multigeneration households, they still lacked sufficient individual‐level support to adhere to home exercise programs. The absence of, or poor social support for exercise requires further investigation. It is possible that limited awareness of exercise benefits for fall prevention and reduced concern about falls during exercise may have inadvertently constrained their activities.^36^ Further, family members in Singapore may have limited time to supervise exercise, due to other work or social obligations (i.e., needing to provide care to both the young and older generations).^38^ Hence, families need to be better equipped to support and complement caregivers/health professionals to facilitate exercise adherence.

Surprisingly, self‐efficacy for exercise was negatively associated with exercise adherence, contradicting previous research.^29^, ^30^, ^39^, ^40^, ^41^ This could be due to overestimation of task demands when requirements are unclear, while underestimation leads to underconfidence.^42^ Ambiguity about the nature of the activity moderates the self‐efficacy–performance relationship, with a negative relation at high ambiguity and a positive relation at low ambiguity.^43^ High self‐efficacy individuals may allocate fewer resources, decreasing performance.^44^ Low self‐efficacy individuals may receive more social support for exercise, positively impacting adherence. Cultural values on exercise in older age, perceptions, health beliefs, and prioritizing family interests contribute to the reported low self‐efficacy.^45^, ^46^, ^47^ Healthcare providers should provide information, education,^48^ and address doubts early in exercise interventions to alleviate negative attitudes and beliefs.

Gender, level of education, and performance on the 30 s chair stand test did not appear to predict exercise adherence. This result is surprising, given that previous studies reported that older adults who were female,^49^ with higher education levels (>high school),^50^ and poor functional performance,^11^ were more likely to engage with/adhere to exercise activities to prevent falls. Further investigation is required to determine the relative importance of these and other potential predictors of adherence that provide valuable information about which patients are more/less likely to adhere to falls prevention exercise programs, and thereafter maintain ongoing participation in exercise.

Higher exercise adherence correlates with increased daily step count, suggesting a potential connection, but causality cannot be inferred due to the study's cross‐sectional design. Prior research indicates that low adherence hampers exercise effectiveness, impacting physical function/performance.^51^ As our participants were clinical populations with low physical activity levels, they would benefit most from increasing exercise. Clinicians should offer optimal guidance, including setting exercise goals, to encourage ongoing participation.

### 4.1 Limitations

This study has several limitations. Firstly, the main outcome relied on self‐report exercise diaries, which may introduce bias towards over‐reporting.^52^, ^53^ However, diaries offer valuable information on exercise type, individual sessions, and contextual factors that accelerometry cannot capture. Additionally, self‐report diaries are simple and cost‐effective methods for prospective data collection.^54^ Secondly, caution is warranted in interpreting the findings due to the small sample size. Recruitment during the COVID‐19 pandemic and restrictions on community activities (such as research) in Singapore limited participation. This small sample size may have prevented us from detecting some significant relationships. Therefore, we acknowledge the potential for chance findings and emphasize the need for a cautious interpretation of the results. Thirdly, convenience sampling introduces the possibility of selection bias. The findings are limited to older adults at risk of falling and may not be extrapolated to other populations (e.g., institutionalized, healthy people).

This study provides evidence for the real‐world adherence patterns of older adults at risk of falling undergoing standard care at hospital‐based outpatient clinics as well as factors predicting home exercise adherence. Older adults in Singapore who took more medications and had better social support, but low self‐efficacy, were most likely to adhere to prescribed home exercise programs for falls prevention. Various factors, including individual characteristics (e.g., medication intake, use of a walking aid), program features (e.g., functional exercises), physical capability (e.g., strength), and psychosocial qualities (e.g., self‐efficacy, social support) may serve as indicators of poor exercise adherence. Further work is needed to explore the interaction of these factors and other predictors to enhance adherence to exercise recommendations. This calls for a larger sample size to corroborate and broaden our findings, alongside a recognition of the significance of replication studies in validating our outcomes.

## AUTHOR CONTRIBUTIONS

Concept and design: Bernadine Teng, Sandra G. Brauer, Anna L. Hatton, and Sjaan R. Gomersall. Data collection: Bernadine Teng. Data analysis: Bernadine Teng, Sandra G. Brauer, Asaduzzaman Khan, and Sjaan R. Gomersall. All authors reviewed and gave approval for the final version.

## FUNDING INFORMATION

Not applicable.

## CONFLICT OF INTEREST STATEMENT

None declared.

## ETHICS STATEMENT

Ethic approval for the study was gained from The Singapore National Healthcare Group ‐ Domain Specific Review Board (2018/01372) and The University of Queensland Human Research Ethics Committee (HREC/2019001991).

## CONSENT

Participants provided consent for the anonymous publication of data.

## Supporting information


Appendix S1.

Appendix S2.
