# Supplementary material for: Predictors of real‐world adherence to prescribed home exercise in older patients with a risk of falling: A prospective observational study
Source: Aging Med (Milton). 2023 Sep 27;6(4):361–9. doi: 10.1002/agm2.12270 (PMC10792326; doi:10.1002/agm2.12270)
Supplement: Supplementary file 1 — Appendix S1. [file AGM2-6-361-s001.pdf]

Appendix 1. List of potential predictor variables measured

No	Potential Variables	Source	Scoring system
	Sociodemographic		
1	Age	Self-reported	Years
2	Body mass index	Clinical record	kg/m2
3	Gender	Clinical record	Female versus Male
4	Marital status	Clinical record	Yes versus No
5	Ethnicity	Self-reported	4 categories: Chinese/Indian/Malay/Other
6	Education level	Self-reported	4 categories: No formal education/Primary/Secondary/Tertiary
7	Living situation	Self-reported	3 categories: Alone/With spouse or children/With other family members/friends
8	Multigeneration household (≥3)	Self-reported	Yes versus No
	Clinical characteristics		
1	Visual impaired	Self-reported	Yes versus No
2	Hearing impaired	Self-reported	Yes versus No
3	Functional mobility	Self-reported	Independent versus non-independent
4	Falls history in the last 12 months	Self-reported	Yes versus No
5	Number of falls	Self-reported	4 categories: None/Once/Twice/Three or more
6	Pain	Self-reported	Yes versus No
7	Use of walking aid	Self-reported	Yes versus No
8	Number of medications	Clinical record	Total number of medications taken
9	Number of chronic conditions	Clinical record	Total number of chronic conditions
	Psychosocial		
1	Short Falls Efficacy Scale-International	Self-reported	Item score: 7-28
2	Self-efficacy for Exercise Scale	Self-reported	Item score: 0-90
3	Social support for Exercise Behaviours	Self-reported	Item score: 0-100
4	Short R-UCLA Loneliness Scale	Self-reported	Item score: 3-9
	Physical performance		
1	30-second Chair Stand test	Scored by therapist	Repetitions
2	Timed Up and Go test	Scored by therapist	Seconds
3	Phone-FITT	Self-reported	Item score: 0-209
	Inervention-specific		

1	Number of different home exercises prescribed	Clinical record	Total number of exercises over 6 weeks
2	Frequency of onsite follow-up	Clinical record	Total number of visits over 6 weeks
3	Time between T0 and the first onsite follow-up	Clinical record	Week(s)
4	Balance component in prescribed exercise	Clinical record	Yes versus No
5	Strength component in prescribed exercise	Clinical record	Yes versus No
6	Functional component in prescribed exercise	Clinical record	Yes versus No
7	Combined exercises prescribed	Clinical record	Yes versus No
