# Supplementary material for: Predictors of real‐world adherence to prescribed home exercise in older patients with a risk of falling: A prospective observational study
Source: Aging Med (Milton). 2023 Sep 27;6(4):361–9. doi: 10.1002/agm2.12270 (PMC10792326; doi:10.1002/agm2.12270)

Appendix 2 Independent -Sample *t* Test and chi-square test

Group Statistics					
	Group	N	Mean	Std. Deviation	Std. Error Mean
Age	Excluded	24	78.67	6.357	1.298
	Included	44	79.00	7.048	1.063
BMI_kg/m2	Excluded	23	25.035	7.3093	1.5241
	Included	42	24.895	5.3956	.8326
Household generation	Excluded	24	1.46	1.250	.255
	Included	43	1.81	1.258	.192
Number of medications	Excluded	21	5.24	3.463	.756
	Included	44	5.09	2.956	.446
Social support for exercise	Excluded	22	26.09	7.867	1.677
	Included	44	30.73	12.080	1.821
Self-efficacy for exercise	Excluded	23	35.22	19.895	4.148
	Included	44	36.05	23.678	3.570
Falls efficacy scale international	Excluded	24	15.33	6.742	1.376
	Included	44	18.57	6.021	.908
3 -item loneliness scale	Excluded	23	4.13	1.290	.269
	Included	44	4.18	1.435	.216
pre_TUG (s)	Excluded	20	19.5050	16.51760	3.69345
	Included	34	25.3257	20.64607	3.54077
pre_30sChair_Stand (reps)	Excluded	9	7.22	6.496	2.165
	Included	44	6.48	4.683	.706
Phone-Fitt	Excluded	22	24.0227	14.11051	3.00837
	Included	44	24.2670	16.17756	2.43886

Independent Samples Test

		Levene's Test for Equality of Variances		t-test for Equality of Means						
									95% Confidence Interval of the Difference	
		F	Sig.	t	df	Sig. (2-tailed)	Mean Difference	Std. Error Difference	Lower	Upper
age	Equal variances assumed	.568	.454	-.193	66	.848	-.333	1.729	-3.786	3.119
	Equal variances not assumed			-.199	51.743	.843	-.333	1.677	-3.699	3.032
BMI_kg/m2	Equal variances assumed	.003	.957	.088	63	.930	.1395	1.5907	-3.0392	3.3182
	Equal variances not assumed			.080	35.398	.936	.1395	1.7367	-3.3847	3.6638
household generation	Equal variances assumed	.092	.762	-1.112	65	.270	-.356	.320	-.995	.283
	Equal variances not assumed			-1.114	47.963	.271	-.356	.319	-.998	.286
Number of medications	Equal variances assumed	1.006	.320	.178	63	.860	.147	.829	-1.509	1.804
	Equal variances not assumed			.168	34.393	.868	.147	.877	-1.635	1.929
Social support for exercise	Equal variances assumed	2.299	.134	-1.632	64	.108	-4.636	2.841	-10.311	1.039
	Equal variances not assumed			-1.873	59.388	.066	-4.636	2.476	-9.590	.317
Self efficacy for exercise	Equal variances assumed	.336	.564	-.143	65	.887	-.828	5.781	-12.374	10.718
	Equal variances not assumed			-.151	52.042	.880	-.828	5.473	-11.810	10.154
Falls efficacy scale international	Equal variances assumed	1.880	.175	-2.029	66	.046	-3.235	1.594	-6.417	-.052
	Equal variances not assumed			-1.962	43.012	.056	-3.235	1.649	-6.559	.090
3 item loneliness scale	Equal variances assumed	.045	.832	-.144	65	.886	-.051	.357	-.764	.662
	Equal variances not assumed			-.149	49.146	.882	-.051	.345	-.745	.642
pre_TUG (s)	Equal variances assumed	1.709	.197	-1.074	52	.288	-5.82074	5.42201	-16.70079	5.05932
	Equal variances not assumed			-1.138	47.077	.261	-5.82074	5.11650	-16.11337	4.47190
pre_30sChair_Stand (reps)	Equal variances assumed	1.961	.167	.406	51	.686	.745	1.833	-2.935	4.425
	Equal variances not assumed			.327	9.771	.750	.745	2.277	-4.346	5.836
PhoneFit	Equal variances assumed	.341	.561	-.060	64	.952	-.24432	4.05505	-8.34522	7.85658
	Equal variances not assumed			-.063	47.627	.950	-.24432	3.87277	-8.03262	7.54398

Crosstabs

Case Processing Summary

	Cases					
	Valid		Missing		Total	
	N	Percent	N	Percent	N	Percent
gender * Group	68	100.0%	0	0.0%	68	100.0%

gender * Group Crosstabulation

Count

		Group		Total
		Excluded	Included	
gender	male	12	19	31
	female	12	25	37
Total		24	44	68

Chi-Square Tests

	Value	df	Asymptotic Significance (2-sided)	Exact Sig. (2-sided)	Exact Sig. (1-sided)
Pearson Chi-Square	.291[a]	1	.590		
Continuity Correction[b]	.081	1	.776		
Likelihood Ratio	.291	1	.590		
Fisher's Exact Test				.619	.387
Linear-by-Linear Association	.287	1	.592		
N of Valid Cases	68				

a. 0 cells (0.0%) have expected count less than 5. The minimum expected count is 10.94.

b. Computed only for a 2x2 table

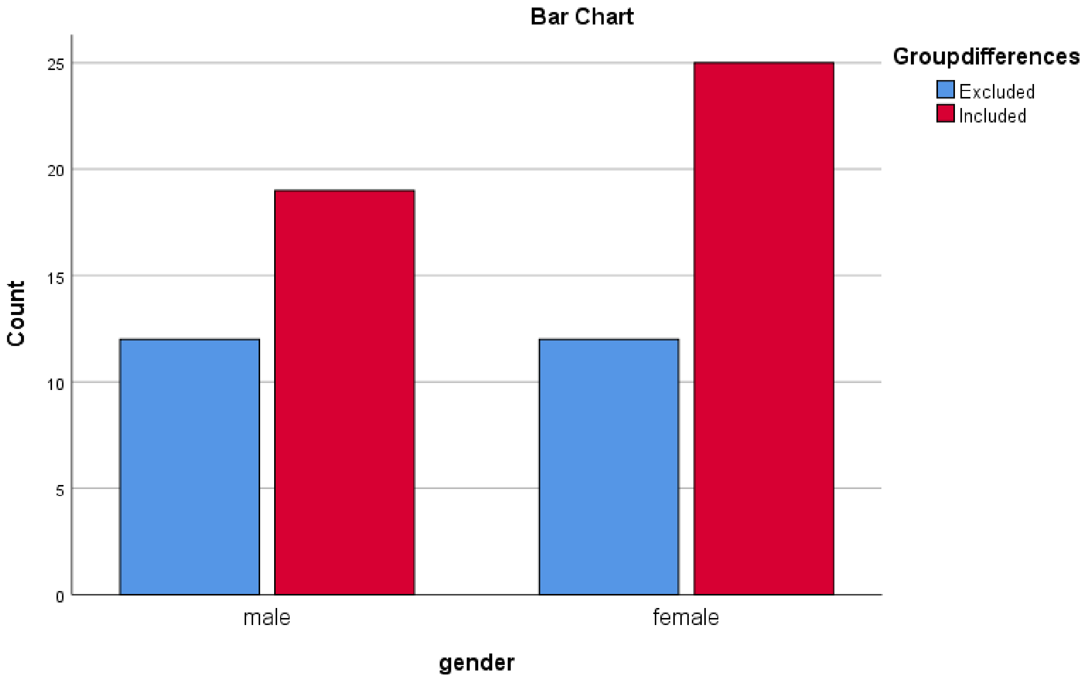

Crosstabs

Case Processing Summary

	Cases					
	Valid		Missing		Total	
	N	Percent	N	Percent	N	Percent
race * Group	68	100.0%	0	0.0%	68	100.0%

race * Group Crosstabulation

Count

		Group		Total
		Excluded	Included	
race	chinese	19	35	54
	malay	3	6	9
	indian	2	2	4
	other	0	1	1
Total		24	44	68

Chi-Square Tests

	Value	df	Asymptotic Significance (2-sided)
Pearson Chi-Square	.940[a]	3	.816
Likelihood Ratio	1.248	3	.741
Linear-by-Linear Association	.001	1	.982
N of Valid Cases	68		

a. 5 cells (62.5%) have expected count less than 5. The minimum expected count is .35.

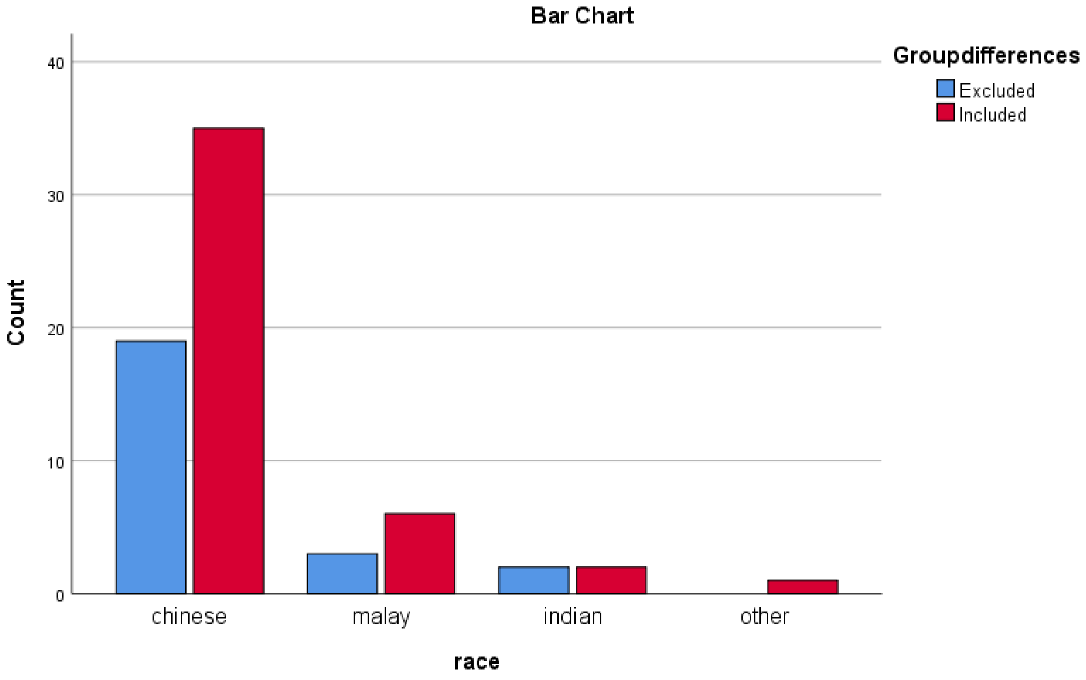

Crosstabs

Case Processing Summary

	Cases					
	Valid		Missing		Total	
	N	Percent	N	Percent	N	Percent
Education level * Group	66	97.1%	2	2.9%	68	100.0%

Education level * Group Crosstabulation

Count

		Group		
		Excluded	Included	Total
Education level	never study	5	14	19
	primary	4	10	14
	secondary	7	10	17

	tertiary	6	10	16
Total		22	44	66

Chi-Square Tests

	Value	df	Asymptotic Significance (2-sided)
Pearson Chi-Square	1.159[a]	3	.763
Likelihood Ratio	1.163	3	.762
Linear-by-Linear Association	.821	1	.365
N of Valid Cases	66		

a. 1 cells (12.5%) have expected count less than 5. The minimum expected count is 4.67.

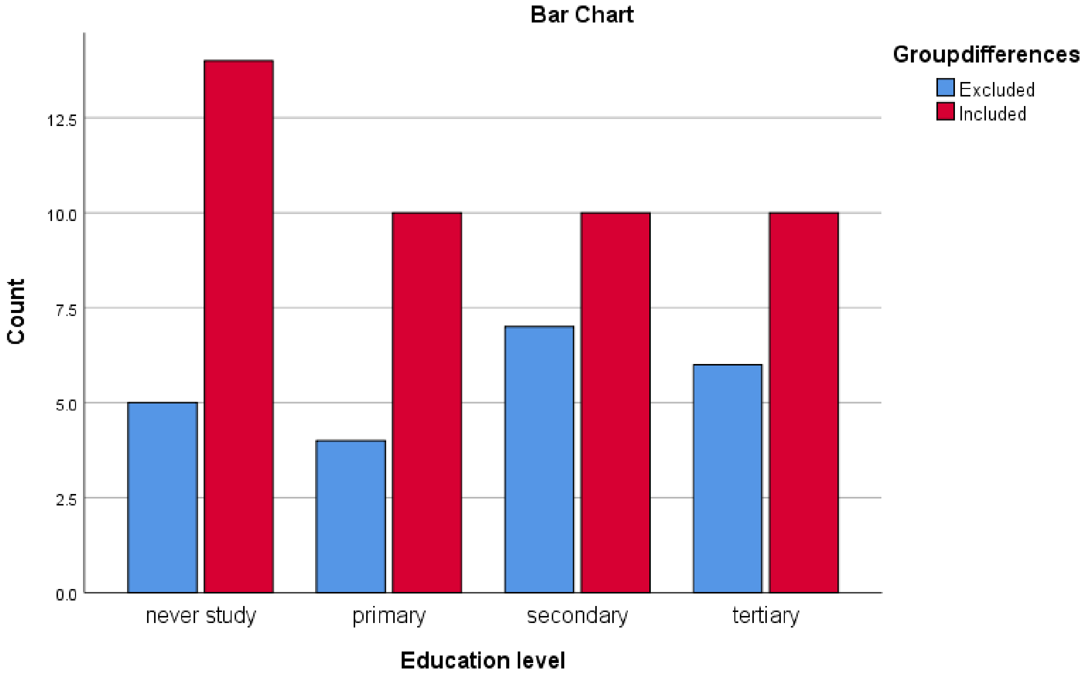

Crosstabs

Case Processing Summary

	Cases					
	Valid		Missing		Total	
	N	Percent	N	Percent	N	Percent
marital status * Group	68	100.0%	0	0.0%	68	100.0%

marital status * Group Crosstabulation

Count

		Group		
		Excluded	Included	Total
marital status	married	21	43	64
	never married	3	1	4
Total		24	44	68

Chi-Square Tests

	Value	df	Asymptotic Significance (2-sided)	Exact Sig. (2-sided)	Exact Sig. (1-sided)
Pearson Chi-Square	2.934[a]	1	.087		
Continuity Correction[b]	1.377	1	.241		
Likelihood Ratio	2.795	1	.095		
Fisher's Exact Test				.122	.122
Linear-by-Linear Association	2.891	1	.089		
N of Valid Cases	68				

a. 2 cells (50.0%) have expected count less than 5. The minimum expected count is 1.41.

b. Computed only for a 2x2 table

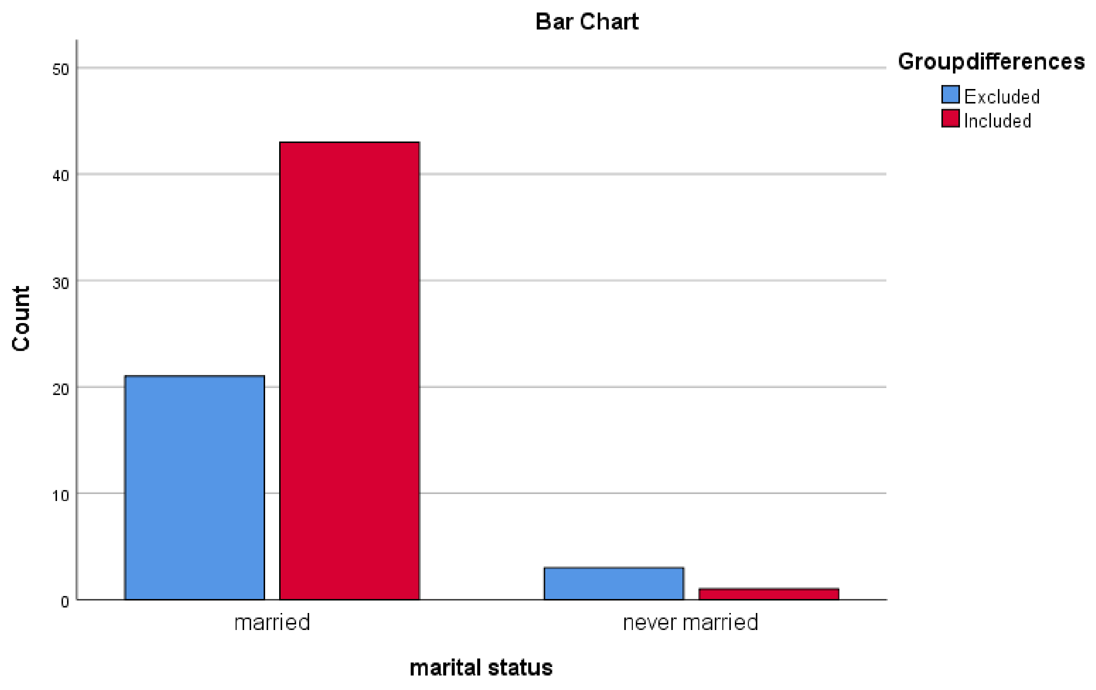

Crosstabs

Case Processing Summary

	Cases					
	Valid		Missing		Total	
	N	Percent	N	Percent	N	Percent
living situation * Group	68	100.0%	0	0.0%	68	100.0%

living situation * Group Crosstabulation

Count

		Group		Total
		Excluded	Included	
living situation	home alone	2	3	5
	Living with spouse/children/maid	18	36	54
	Other-living with sibilings/grandson/niece	4	5	9
Total		24	44	68

Chi-Square Tests

	Value	df	Asymptotic Significance (2-sided)
Pearson Chi-Square	.469[a]	2	.791
Likelihood Ratio	.459	2	.795
Linear-by-Linear Association	.108	1	.742
N of Valid Cases	68		

a. 3 cells (50.0%) have expected count less than 5. The minimum expected count is 1.76.

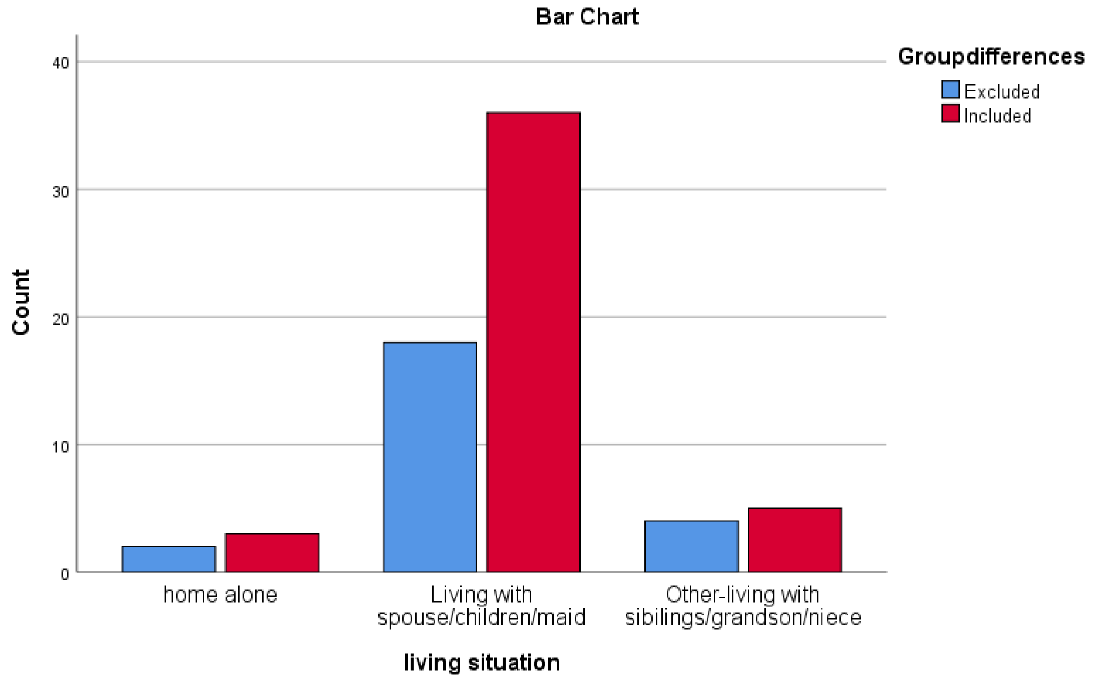

Crosstabs

Case Processing Summary

	Cases					
	Valid		Missing		Total	
	N	Percent	N	Percent	N	Percent
visual impaired * Group	68	100.0%	0	0.0%	68	100.0%

visual impaired * Group Crosstabulation

Count

		Group		Total
		Excluded	Included	
visual impaired	yes	3	15	18
	no	21	29	50
Total		24	44	68

Chi-Square Tests

	Value	df	Asymptotic Significance (2-sided)	Exact Sig. (2-sided)	Exact Sig. (1-sided)

Pearson Chi-Square	3.719[a]	1	.054		
Continuity Correction[b]	2.693	1	.101		
Likelihood Ratio	4.048	1	.044		
Fisher's Exact Test				.083	.047
Linear-by-Linear Association	3.665	1	.056		
N of Valid Cases	68				

a. 0 cells (0.0%) have expected count less than 5. The minimum expected count is 6.35.

b. Computed only for a 2x2 table

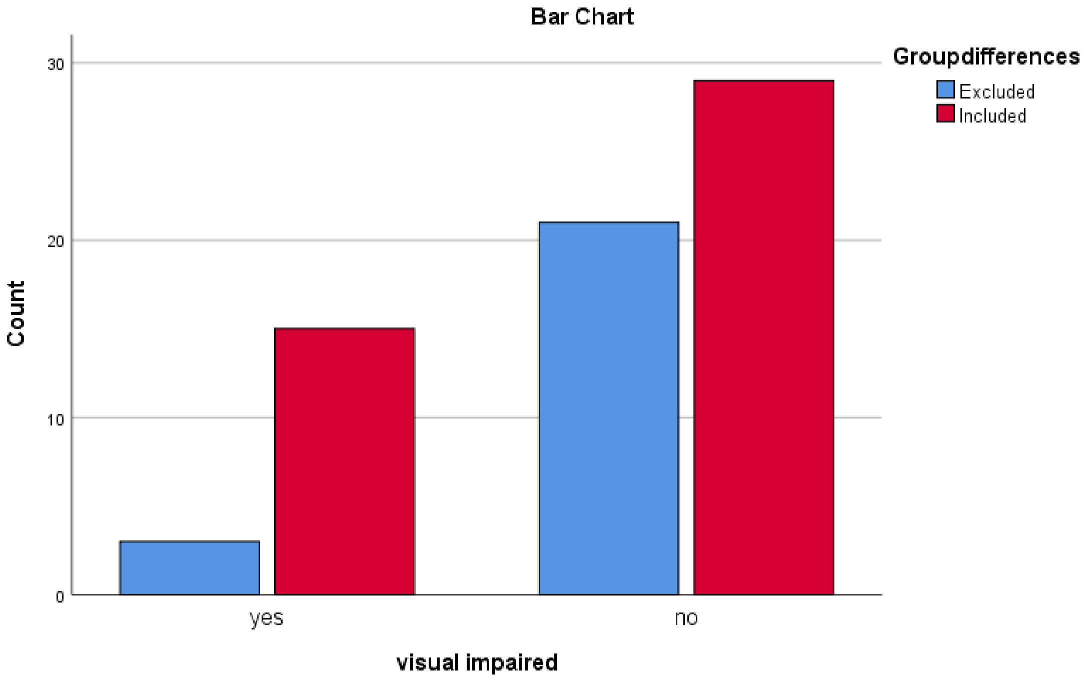

Crosstabs

Case Processing Summary

	Cases					
	Valid		Missing		Total	
	N	Percent	N	Percent	N	Percent
hearing impaired * Group	68	100.0%	0	0.0%	68	100.0%

hearing impaired * Group Crosstabulation

Count

		Group		
		Excluded	Included	Total
hearing impaired	yes	4	13	17
	no	20	31	51
Total		24	44	68

Chi-Square Tests

	Value	df	Asymptotic Significance (2-sided)	Exact Sig. (2-sided)	Exact Sig. (1-sided)
Pearson Chi-Square	1.374[a]	1	.241		
Continuity Correction[b]	.773	1	.379		
Likelihood Ratio	1.438	1	.230		
Fisher's Exact Test				.380	.191
Linear-by-Linear Association	1.354	1	.245		
N of Valid Cases	68				

a. 0 cells (0.0%) have expected count less than 5. The minimum expected count is 6.00.

b. Computed only for a 2x2 table

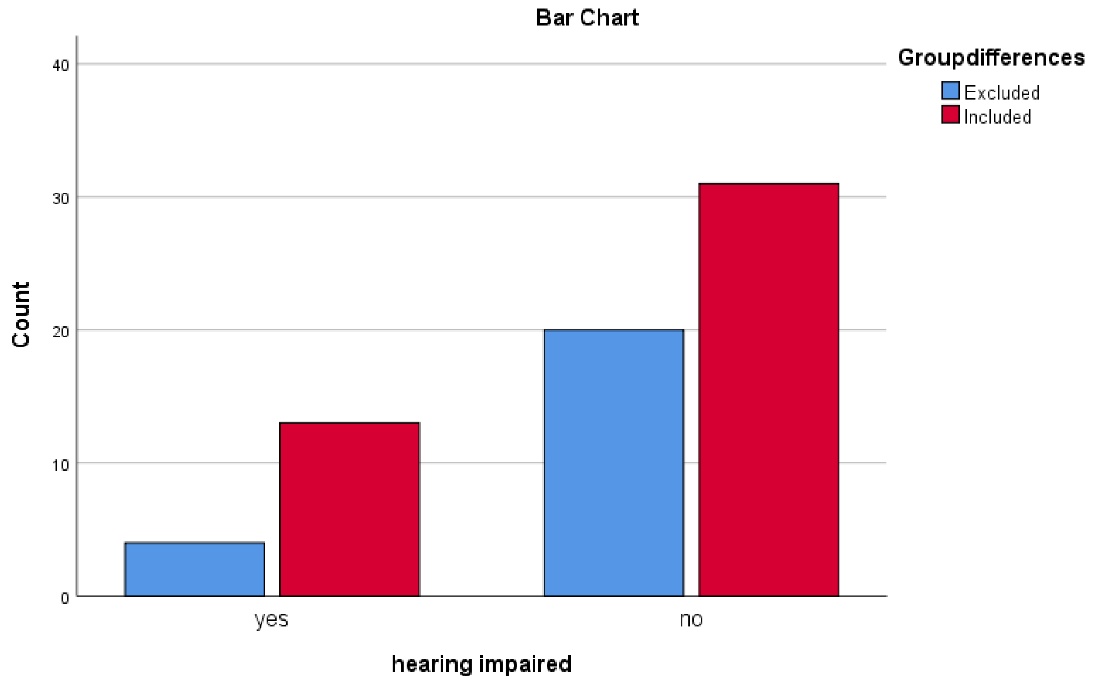

Crosstabs

Case Processing Summary

	Cases					
	Valid		Missing		Total	
	N	Percent	N	Percent	N	Percent
function * Group	68	100.0%	0	0.0%	68	100.0%

function * Group Crosstabulation

Count

		Group		
		Excluded	Included	Total
function	independent	20	32	52
	nonindependent	4	12	16
Total		24	44	68

Chi-Square Tests

	Value	df	Asymptotic Significance (2-sided)	Exact Sig. (2-sided)	Exact Sig. (1-sided)
Pearson Chi-Square	.971[a]	1	.324		
Continuity Correction[b]	.471	1	.493		
Likelihood Ratio	1.010	1	.315		
Fisher's Exact Test				.384	.250
Linear-by-Linear Association	.957	1	.328		
N of Valid Cases	68				

a. 0 cells (0.0%) have expected count less than 5. The minimum expected count is 5.65.

b. Computed only for a 2x2 table

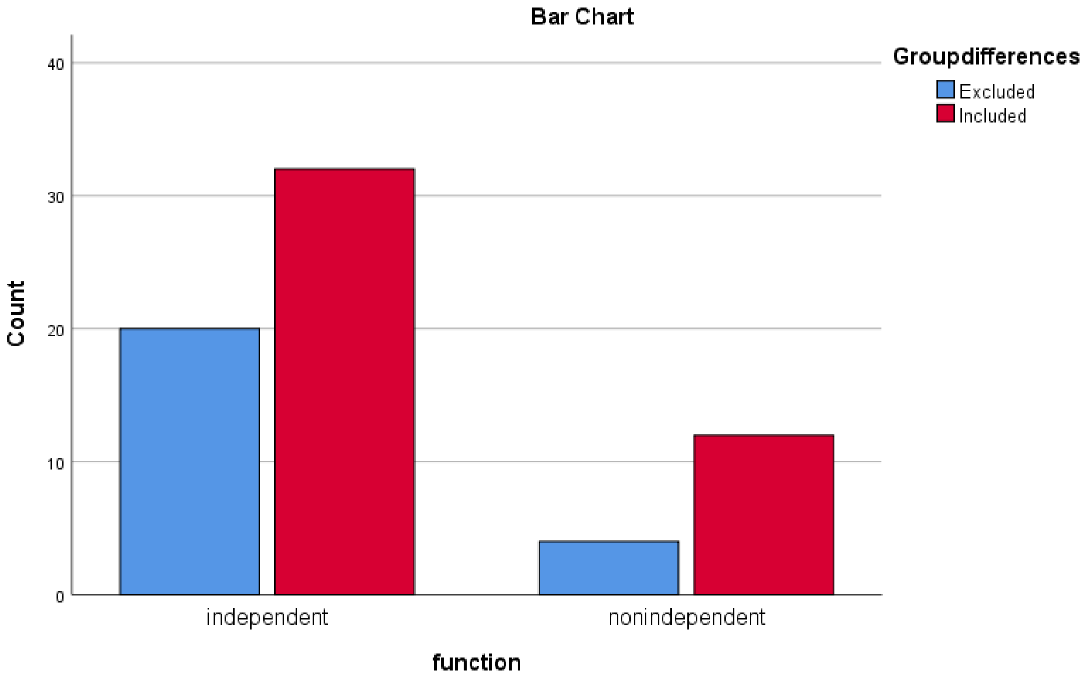

Crosstabs

Case Processing Summary

	Cases					
	Valid		Missing		Total	
	N	Percent	N	Percent	N	Percent
falls history * Group	67	98.5%	1	1.5%	68	100.0%

falls history * Group Crosstabulation

Count

		Group		Total
		Excluded	Included	
falls history	yes	20	32	52
	no	3	12	15
Total		23	44	67

Chi-Square Tests

	Value	df	Asymptotic Significance (2-sided)	Exact Sig. (2-sided)	Exact Sig. (1-sided)
Pearson Chi-Square	1.760[a]	1	.185		
Continuity Correction[b]	1.036	1	.309		
Likelihood Ratio	1.882	1	.170		
Fisher's Exact Test				.230	.154
Linear-by-Linear Association	1.734	1	.188		
N of Valid Cases	67				

a. 0 cells (0.0%) have expected count less than 5. The minimum expected count is 5.15.

b. Computed only for a 2x2 table

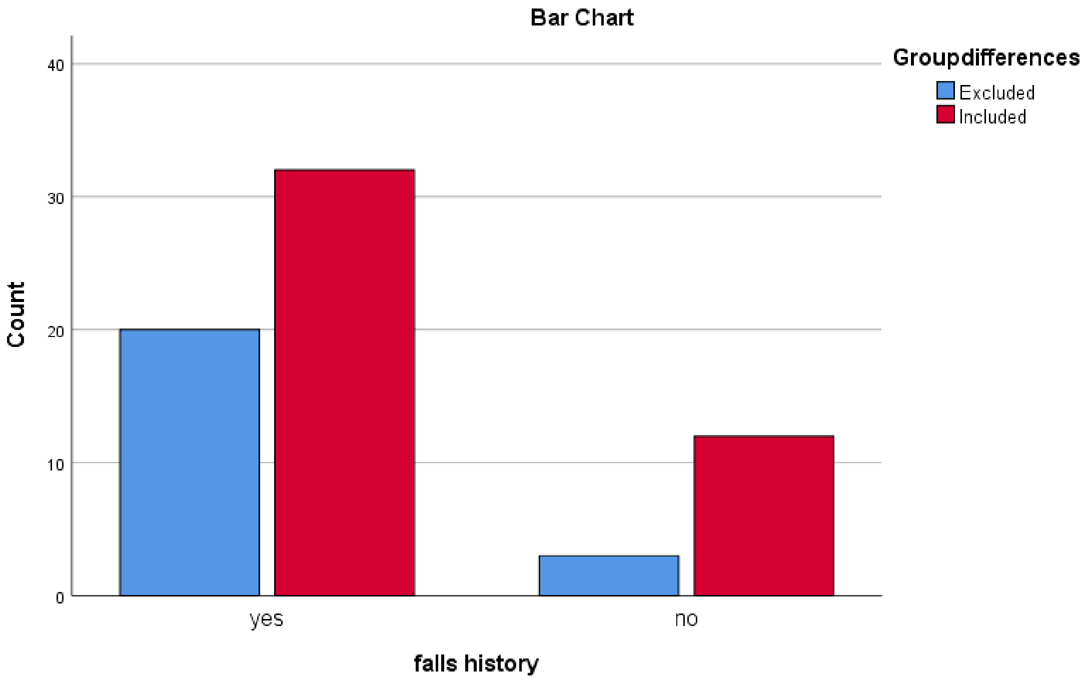

Crosstabs

Case Processing Summary

	Cases					
	Valid		Missing		Total	
	N	Percent	N	Percent	N	Percent
Number of falls * Group	66	97.1%	2	2.9%	68	100.0%

Number of falls * Group Crosstabulation

Count

		Group		
		Excluded	Included	Total
Number of falls	none	2	12	14
	once	7	20	27
	twice	4	3	7
	three or more	9	9	18
Total		22	44	66

Chi-Square Tests

	Value	df	Asymptotic Significance (2-sided)
Pearson Chi-Square	6.988[a]	3	.072
Likelihood Ratio	7.119	3	.068
Linear-by-Linear Association	5.896	1	.015
N of Valid Cases	66		

a. 3 cells (37.5%) have expected count less than 5. The minimum expected count is 2.33.

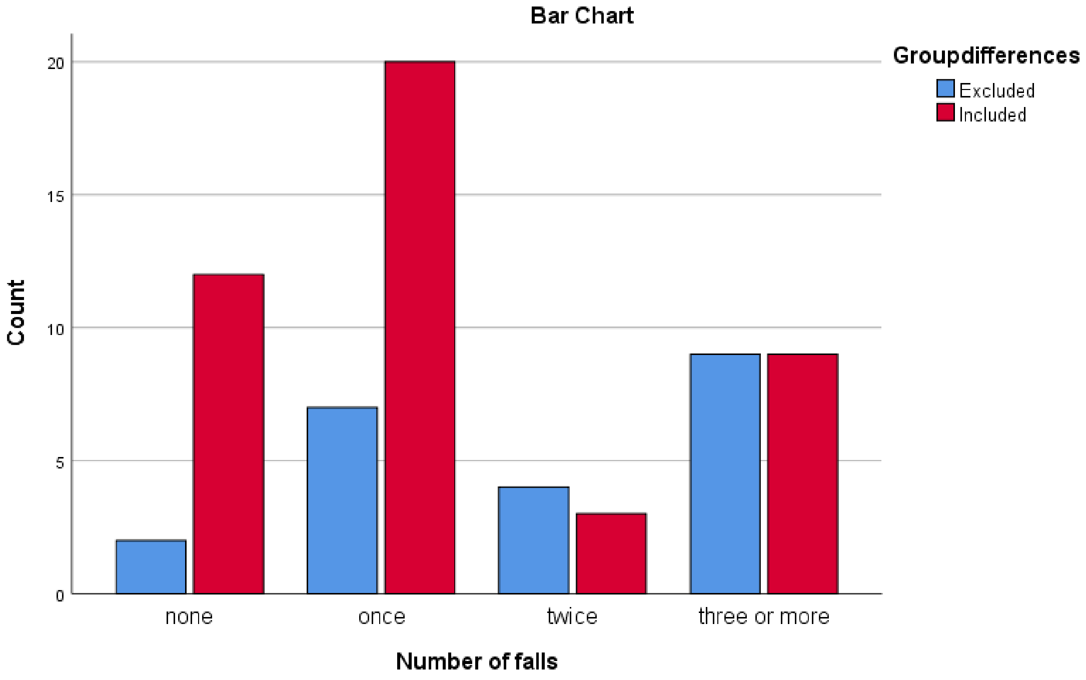

Crosstabs

Case Processing Summary

	Cases					
	Valid		Missing		Total	
	N	Percent	N	Percent	N	Percent
Pain * Group	61	89.7%	7	10.3%	68	100.0%

Pain * Group Crosstabulation

Count

		Group		
		Excluded	Included	Total
Pain	yes	6	18	24
	no	11	26	37
Total		17	44	61

Chi-Square Tests

	Value	df	Asymptotic Significance (2-sided)	Exact Sig. (2-sided)	Exact Sig. (1-sided)
Pearson Chi-Square	.162[a]	1	.687		
Continuity Correction[b]	.012	1	.912		
Likelihood Ratio	.163	1	.686		
Fisher's Exact Test				.775	.460
Linear-by-Linear Association	.159	1	.690		
N of Valid Cases	61				

a. 0 cells (0.0%) have expected count less than 5. The minimum expected count is 6.69.

b. Computed only for a 2x2 table

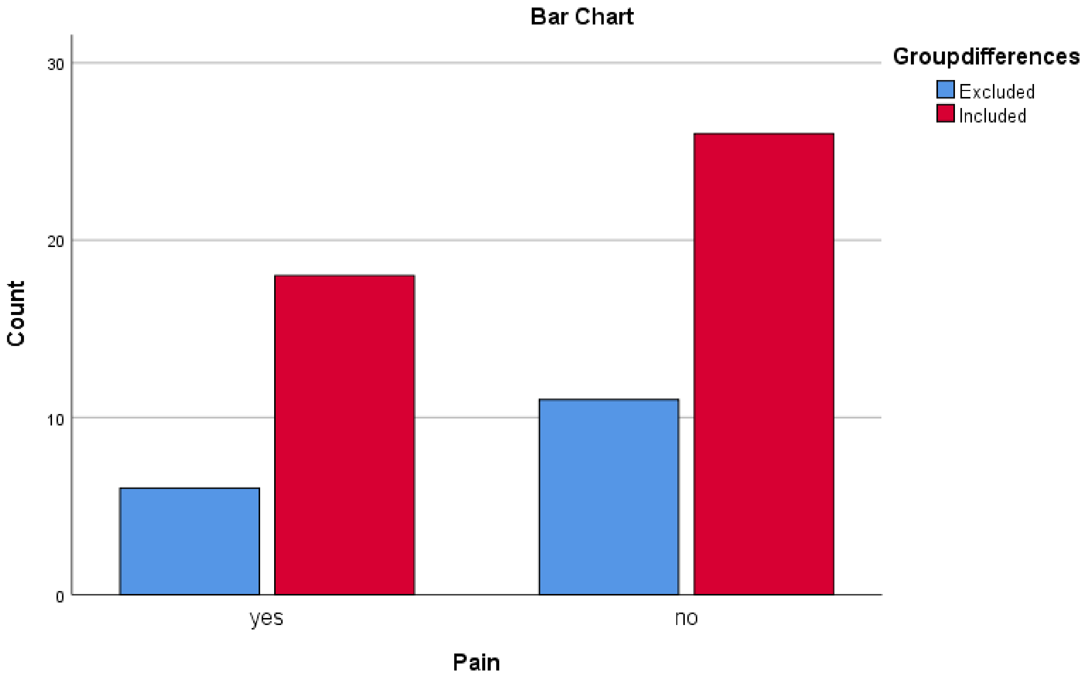

Crosstabs

Case Processing Summary

	Cases					
	Valid		Missing		Total	
	N	Percent	N	Percent	N	Percent
use walking aid * Group	68	100.0%	0	0.0%	68	100.0%

use walking aid * Group Crosstabulation

Count

		Group		
		Excluded	Included	Total
use walking aid	yes	15	21	36
	no	9	23	32
Total		24	44	68

Chi-Square Tests

	Value	df	Asymptotic Significance (2-sided)	Exact Sig. (2-sided)	Exact Sig. (1-sided)
Pearson Chi-Square	1.360[a]	1	.243		
Continuity Correction[b]	.832	1	.362		
Likelihood Ratio	1.372	1	.242		
Fisher's Exact Test				.312	.181
Linear-by-Linear Association	1.340	1	.247		
N of Valid Cases	68				

a. 0 cells (0.0%) have expected count less than 5. The minimum expected count is 11.29.

b. Computed only for a 2x2 table

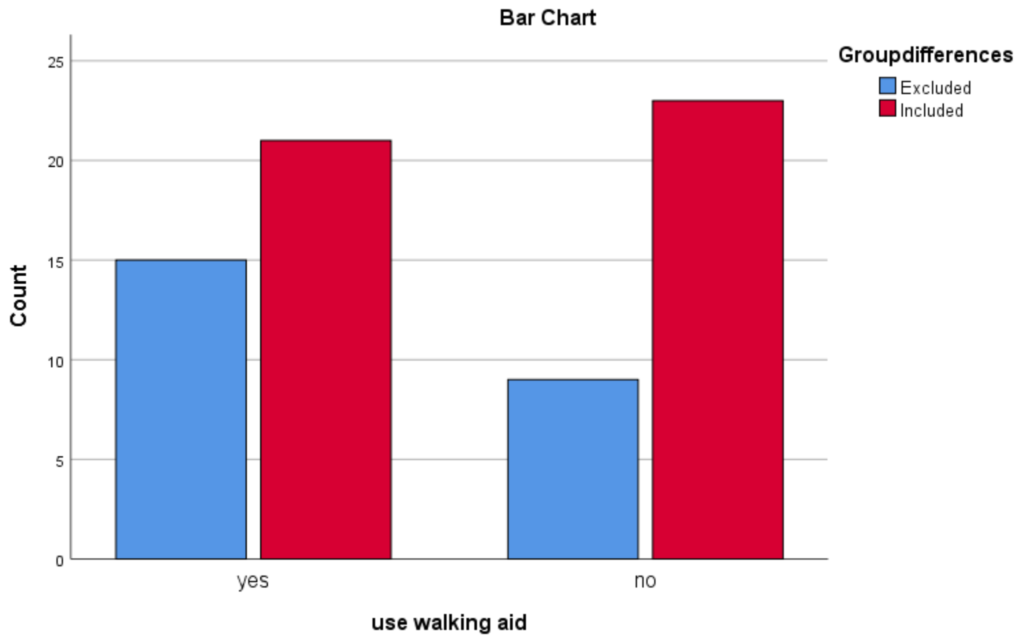

Supplement: Supplementary file 2 — Appendix S2. [file AGM2-6-361-s002.pdf]
